# Synergistic Antibiofilm Effects of Pseudolaric Acid A Combined with Fluconazole against Candida albicans via Inhibition of Adhesion and Yeast-To-Hypha Transition

**DOI:** 10.1128/spectrum.01478-21

**Published:** 2022-03-17

**Authors:** Bin Zhu, Zhen Li, Hongmei Yin, Jun Hu, Yingjun Xue, Guanyi Zhang, Xin Zheng, Weiqin Chen, Xiaobo Hu

**Affiliations:** a Department of Laboratory Medicine, Longhua Hospital, Shanghai University of Traditional Chinese Medicine, Shanghai, People’s Republic of China; Mycology Laboratory, Wadsworth Center

**Keywords:** *Candida albicans*, antibiofilm, fluconazole, pseudolaric acid A, resistance, synergism

## Abstract

Candida albicans biofilms are resistant to several clinical antifungal agents. Thus, it is necessary to develop new antibiofilm intervention measures. Pseudolaric acid A (PAA), a diterpenoid mainly derived from the pine bark of Pseudolarix kaempferi, has been reported to have an inhibitory effect on C. albicans. The primary aim of the current study was to investigate the antibiofilm effect of PAA when combined with fluconazole (FLC) and explore the underlying mechanisms. Biofilm activity was assessed by tetrazolium {XTT [2,3-bis-(2-methoxy-4-nitro-5-sulfophenyl)-2H-tetrazolium-5-carboxanilide salt]} reduction assays. PAA (4 μg/mL) combined with FLC (0.5 μg/mL) significantly inhibited early, developmental, and mature biofilm formation compared with the effect of PAA or FLC alone (*P* < 0.05). Furthermore, PAA (4 μg/mL) combined with FLC (0.5 μg/mL) produced a 56% reduction in C. albicans biofilm adhesion. The combination of PAA (4 μg/mL) and FLC (0.5 μg/mL) also performed well in inhibiting yeast-to-hypha transition. Transcriptome analysis using RNA sequencing and quantitative reverse transcription PCR indicated that the PAA-FLC combination treatment produced a strong synergistic inhibitory effect on the expression of genes involved in adhesion (*ALS1*, *ALS4*, and *ALS2*) and yeast-to-hypha transition (*ECE1*, *PRA1*, and *TEC1*). Notably, PAA, rather than FLC, may have a primary role in suppressing the expression of *ALS1*. In conclusion, these findings demonstrate, for the first time, that the combination of PAA and FLC has an improved antibiofilm effect against the formation of C. albicans biofilms by inhibiting adhesion and yeast-to-hypha transition; this may provide a novel therapeutic strategy for treating C. albicans biofilm-associated infection.

**IMPORTANCE** Biofilms are the primary cause of antibiotic-resistant candida infections associated with medical implants and devices worldwide. Treating biofilm-associated infections is a challenge for clinicians because these infections are intractable and persistent. Candida albicans readily forms extensive biofilms on the surface of medical implants and mucosa. In this study, we demonstrated, for the first time, an inhibitory effect of pseudolaric acid A alone and in combination with fluconazole on C. albicans biofilms. Moreover, pseudolaric acid A in combination with fluconazole exerted an antibiofilm effect through multiple pathways, including inhibition of yeast-to-hypha transition and adhesion. This research not only provides new insights into the synergistic mechanisms of antifungal drug combinations but also brings new possibilities for addressing C. albicans drug resistance.

## INTRODUCTION

Candida albicans, a commensal organism, is normally found on the skin and mucous membrane surfaces of humans. However, when the human microbiota is imbalanced and the immune system impaired, Candida albicans can become pathogenic and cause invasive candidiasis (1). Statistics indicate that at least 250,000 new cases of invasive candidiasis are diagnosed worldwide each year and more than 50,000 people die from invasive candidiasis (2). Candida albicans is the most common pathogen causing invasive candidiasis, accounting for 40 to 50% of cases (3). One crucial factor that is conducive to the pathogenesis of invasive C. albicans candidiasis is biofilm formation, since C. albicans is able to form biofilms readily on both abiotic and biotic surfaces (4). Combating C. albicans biofilms requires the use of antifungal drugs. Antifungal drugs used clinically primarily include the polyene drug classes echinocandins and azoles, of which azoles, especially fluconazole (FLC), are most commonly used in the treatment of C. albicans infections (5, 6). However, biofilms enable genetic resistance to antifungal drugs, including FLC (7), which makes FLC treatment of C. albicans biofilms inefficient. Therefore, there is a critical need to identify new antifungal agents or combination therapies to fight biofilm-related infections.

The utilization of natural products, such as Chinese herbal monomers, offers new prospects for the development of novel drug entities (8). Several phytochemicals have been found to potentially inhibit biofilm formation and, in combination with FLC, exhibit synergistic effects against C. albicans biofilms (9). For example, Dong et al. found that a combination of curcumin derivatives and FLC showed synergistic antifungal activity against C. albicans biofilm formation (10). Pseudolaric acid A (PAA) is a diterpenoid extracted from a traditional Chinese medicine, the root bark of Pseudolarix kaempferi. The root bark of Pseudolarix, known as “Tu-Jin-Pi,” has long been used for the treatment of skin infections in China (11).

Research suggests that PAA has antifungal activity against C. albicans (12). Biofilm formation is one of the primary antifungal mechanisms responsible for the FLC resistance of C. albicans. However, to date, there are no published studies of the effects of PAA alone or in combination with FLC against C. albicans biofilms, and the specific antibiofilm mechanism has not been fully clarified yet.

The aim of the current study was to evaluate and determine the antibiofilm activity of PAA combined with FLC (PAA-FLC) against C. albicans. Furthermore, changes in adherence properties, yeast-to-hypha transition, and the expression levels of specific biofilm-related genes were measured to elucidate the antibiofilm mechanisms of the combination therapy. The ultimate goal of this study was to provide an effective solution to the problem of biofilm-related drug resistance.

## RESULTS

### Effects of PAA or FLC alone against C. albicans biofilm formation.

**FIG 1:**
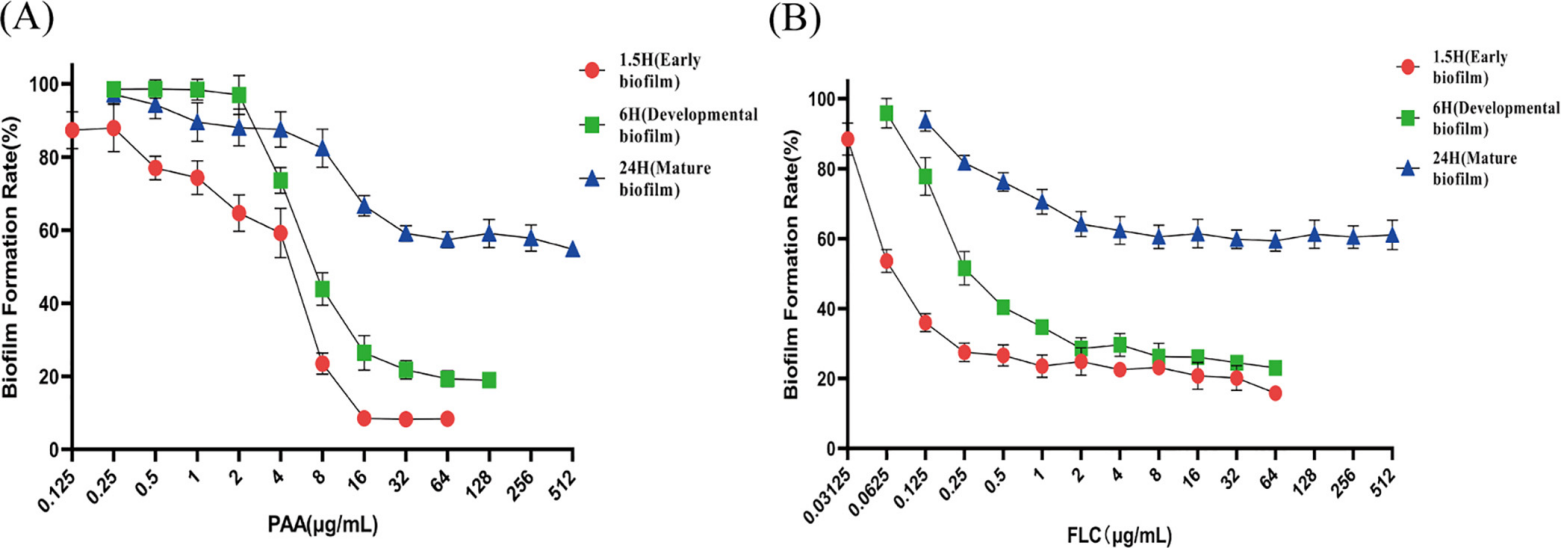
Effect of PAA or FLC against C. albicans biofilm formation at different formation stages. (A) The biofilm formation rate using PAA alone against C. albicans biofilm formation at various time points (1.5, 6, and 24 h). (B) The biofilm formation rate using FLC alone against C. albicans biofilm formation at various time points (1.5, 6, and 24 h). 1.5, 6, and 24 h relate to the time points of drug treatment during biofilm formation. 1.5 h (early biofilm): C. albicans ATCC 90028 cells were allowed to adhere for 1.5 h, and then PAA or FLC alone was added and the cells were incubated for another 24 h at 37°C. 6 h (developmental biofilm): C. albicans ATCC 90028 cells were allowed to adhere for 6 h, and then PAA or FLC alone was added and the cells were incubated for a further 24 h at 37°C. 24 h (mature biofilm): after 24 h of cell adherence to form mature biofilms, C. albicans ATCC 90028 cells were treated with PAA or FLC alone for an additional 24 h at 37°C. Values represent the means ± standard deviation of three replicates.

The process of biofilm formation involves various stages, including the early stage (adhesion), the developmental stage (appearance of hypha), and the mature stage (matrix deposition and hyphal elongation). The antibiofilm effects of PAA or FLC at different stages were evaluated by XTT [2,3-bis-(2-methoxy-4-nitro-5-sulfophenyl)-2H-tetrazolium-5-carboxanilide salt] reduction assays. Based on the results of the XTT reduction assays, the percentage of production at different stages of biofilm formation was calculated and compared with that of the untreated control. During the early biofilm phase (1.5 h), both PAA and FLC produced obvious dose-dependent decreases in C. albicans biofilm formation. The biofilm formation rates were about 20% in 8 μg/mL PAA and 23% in 0.25 μg/mL FLC for C. albicans ATCC 90028 (Fig. 1). At the developmental biofilm stage (6 h), PAA or FLC suppressed biofilm formation in a dose-dependent manner; however, the drug concentration required to suppress biofilm formation was higher than that for the early biofilm stage. At 16 μg/mL, PAA or FLC hindered nearly 80% of biofilm formation by C. albicans (Fig. 1). At the mature stage of biofilm formation (24 h), PAA or FLC alone had no significant antibiofilm activity, even at the highest drug concentration (512 μg/mL) (Fig. 1). Specifically, the biofilm formation rate of early biofilm at 64 μg/mL PAA or FLC was less than 20%; however, the biofilm formation rate of mature biofilm was more than 65% at the same concentration.

### Synergism of PAA and FLC against C. albicans biofilm formation.

**FIG 2:**
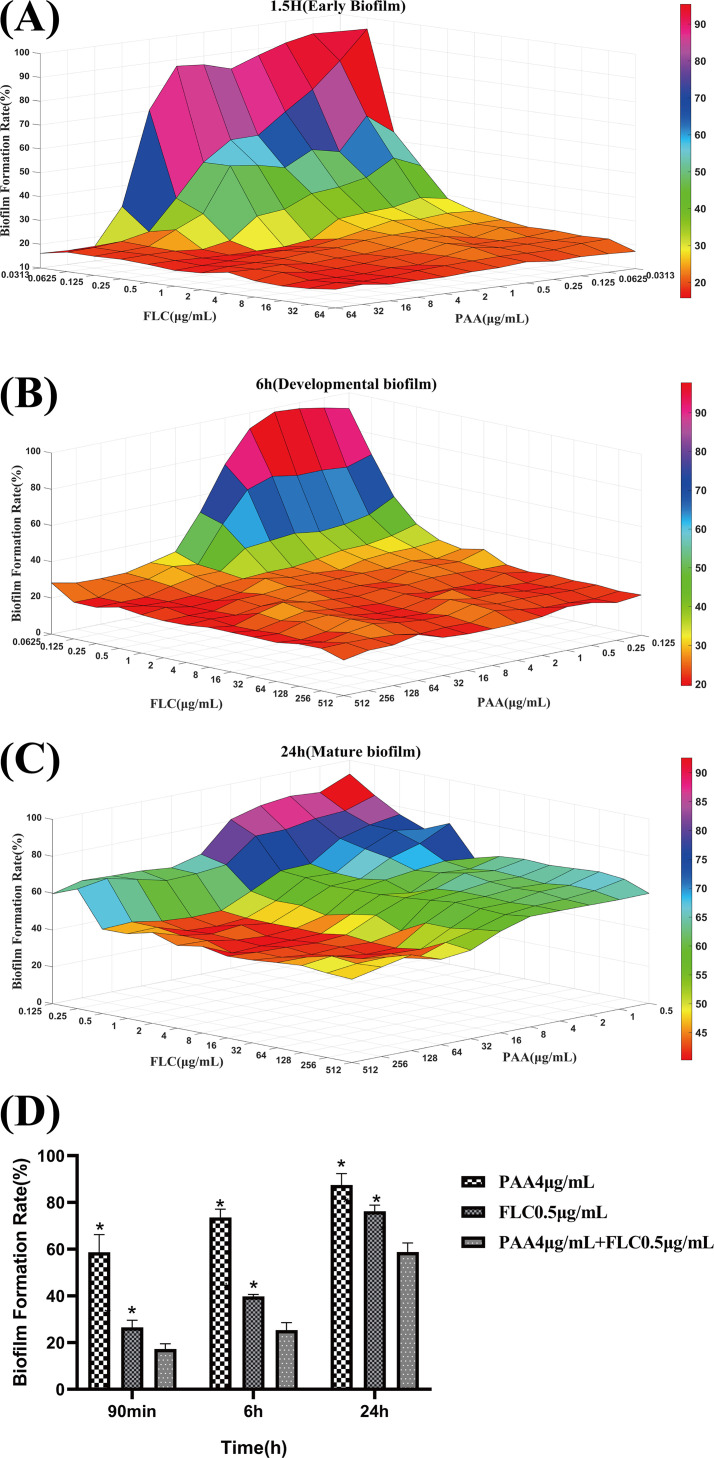
Effect of PAA in combination with FLC against C. albicans biofilm formation at different formation stages. (A) Three-dimensional shaded surface figure of the effect of PAA combined with FLC against C. albicans biofilm formation at 1.5 h (early biofilm). (B) Three-dimensional shaded surface figure of the effect of PAA combined with FLC against C. albicans biofilm formation at 6 h (developmental biofilm). (C) Three-dimensional shaded surface figure of the effect of PAA combined with FLC against C. albicans biofilm formation at 24 h (mature biofilm). Three-dimensional surface plots are provided for the combination of PAA and FLC, in which the *x* and *y* axes indicate the concentrations of PAA and FLC, respectively, and the *z* axis represents the C. albicans biofilm formation rate. For the colored bar on the right, the closer to deep red the color is at the top of the bar, the less effective the drug combination against biofilm formation. (D) Effect of PAA (4 μg/mL) or FLC (0.5 μg/mL) alone on biofilm formation at specific stages (1.5, 6, and 24 h). Values represent the means ± standard deviation of three replicates. *, *P* < 0.05 for the combination of PAA (4 μg/mL) with FLC (0.5 μg/mL) versus either drug alone.

The *in vitro* synergism of PAA in combination with FLC against biofilm formation was examined with checkerboard assays. Compared with the effects of PAA or FLC alone, PAA in combination with FLC exhibited a strong inhibitory effect on biofilm formation in the early, developmental, and mature stages of biofilm formation (Fig. 2A to C). In the early and developmental stages of biofilm formation, with increasing doses of PAA and FLC, the synergistic effect against early and developmental biofilm formation was more evident. PAA (4 μg/mL) in combination with FLC (0.5 μg/mL) showed a significant synergistic effect by reducing biofilm formation by approximately 42% and 49% in the early and developmental biofilm formation stages compared with the reduction observed with 4 μg/mL PAA alone (*P* < 0.05) (Fig. 2D). At the mature biofilm stage (24 h), the effect of the combination treatment was also found to be stronger than that of PAA or FLC alone. For example, FLC (0.5 μg/mL) in combination with PAA at the increasing concentrations of 1, 4, and 32 μg/mL resulted in reductions in biofilm activity of 73%, 58%, and 43%, respectively (Fig. 2C). Interestingly, treatment with the combination of PAA (4 μg/mL) and FLC (0.5 μg/mL) significantly inhibited early, developmental, and mature biofilm formation (*P* < 0.05) compared with PAA or FLC alone (Fig. 2D).

### Effects of PAA and FLC on the growth and metabolic activity of biofilms.

**FIG 3:**
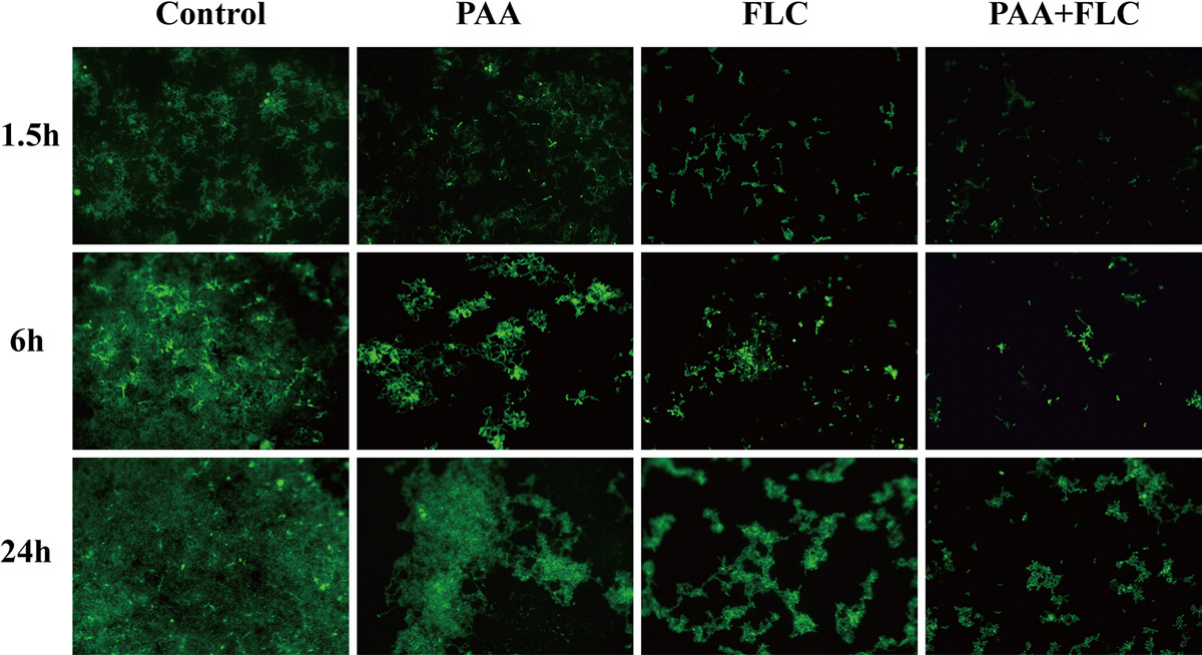
Effect of PAA in combination with FLC on the growth and metabolic activity within the biofilm at different stages (×200 magnification). Adhered biofilm cells were stained with FDA and visualized under a fluorescence microscope. Drug treatments: control (without PAA and FLC), PAA (4 μg/mL), FLC (0.5 μg/mL), and the combination of PAA (4 μg/mL) and FLC (0.5 μg/mL).

The fluorescein diacetate (FDA) method was used to evaluate the growth and metabolic activity of biofilms by observing the number and brightness of fluorescent cells. As shown by the images in Fig. 3, the combination of PAA (4 μg/mL) and FLC (0.5 μg/mL) significantly reduced the growth and cell activity of biofilms in different stages. Biofilms are highly structured microbial communities that are wrapped by an extracellular polymer matrix (13). In the control group without treatment, dense microbial populations composed of yeast cells and mycelia were observed. When FLC (0.5 μg/mL) was combined with PAA (4 μg/mL), the numbers of both planktonic cells and hyphae were lower than the numbers in the drug-free control, and the metabolic activity was also weaker than that in biofilms treated with FLC or PAA alone. This result suggests that PAA in combination with FLC decreased the growth and metabolic activity of biofilms in different formation stages, which is consistent with the results of the XTT reduction assays.

### Potential antibiofilm mechanism studies. (i) PAA combined with FLC decreased adhesion of C. albicans during biofilm formation.

**FIG 4:**
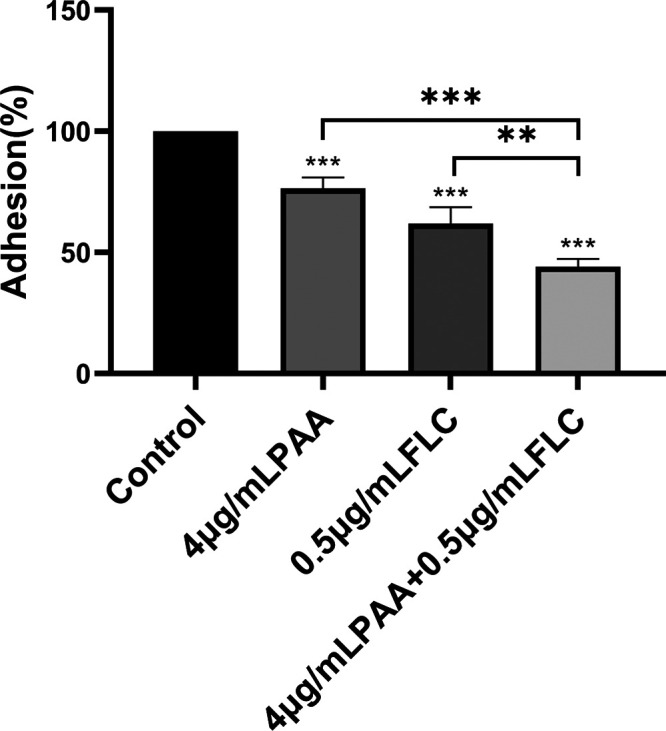
Effect of PAA in combination with FLC on the adhesion of biofilms. The adhesion of biofilms treated with 4 μg/mL PAA, 0.5 μg/mL FLC, and 4 μg/mL PAA plus 0.5 μg/mL FLC was assayed. The data shown are the mean values ± standard deviations from three independent experiments. *, *P* < 0.05, **, *P* < 0.01, and ***, *P* < 0.001 compared with the control group; brackets indicate *P* values for the combination of PAA (4 μg/mL) and FLC (0.5 μg/mL) compared with either drug alone (one-way analysis of variance [ANOVA]).

Since the adhesion of C. albicans is the first step in biofilm formation (14), the effects of PAA and FLC on adhesion were observed. The results of the XTT assay revealed that PAA (4 μg/mL) in combination with FLC (0.5 μg/mL) significantly inhibited biofilm formation. The application of PAA or FLC alone significantly reduced the adhesion rate compared to that in the untreated control; however, the combination of the two drugs further decreased cell adhesion. The adhesion rates of C. albicans treated with PAA (4 μg/mL) or FLC (0.5 μg/mL) alone were 76% and 62%, respectively, while the adhesion rate with the combination of PAA (4 μg/mL) and FLC (0.5 μg/mL) was 44%, which was notably lower than that of PAA alone (*P* < 0.001) or FLC alone (*P* < 0.01) (Fig. 4). This indicates that the combination of PAA and FLC remarkably reduced the adhesion strength of C. albicans during biofilm formation.

### (ii) PAA combined with FLC inhibited yeast-to-hypha transition of C. albicans during biofilm formation.

**FIG 5:**
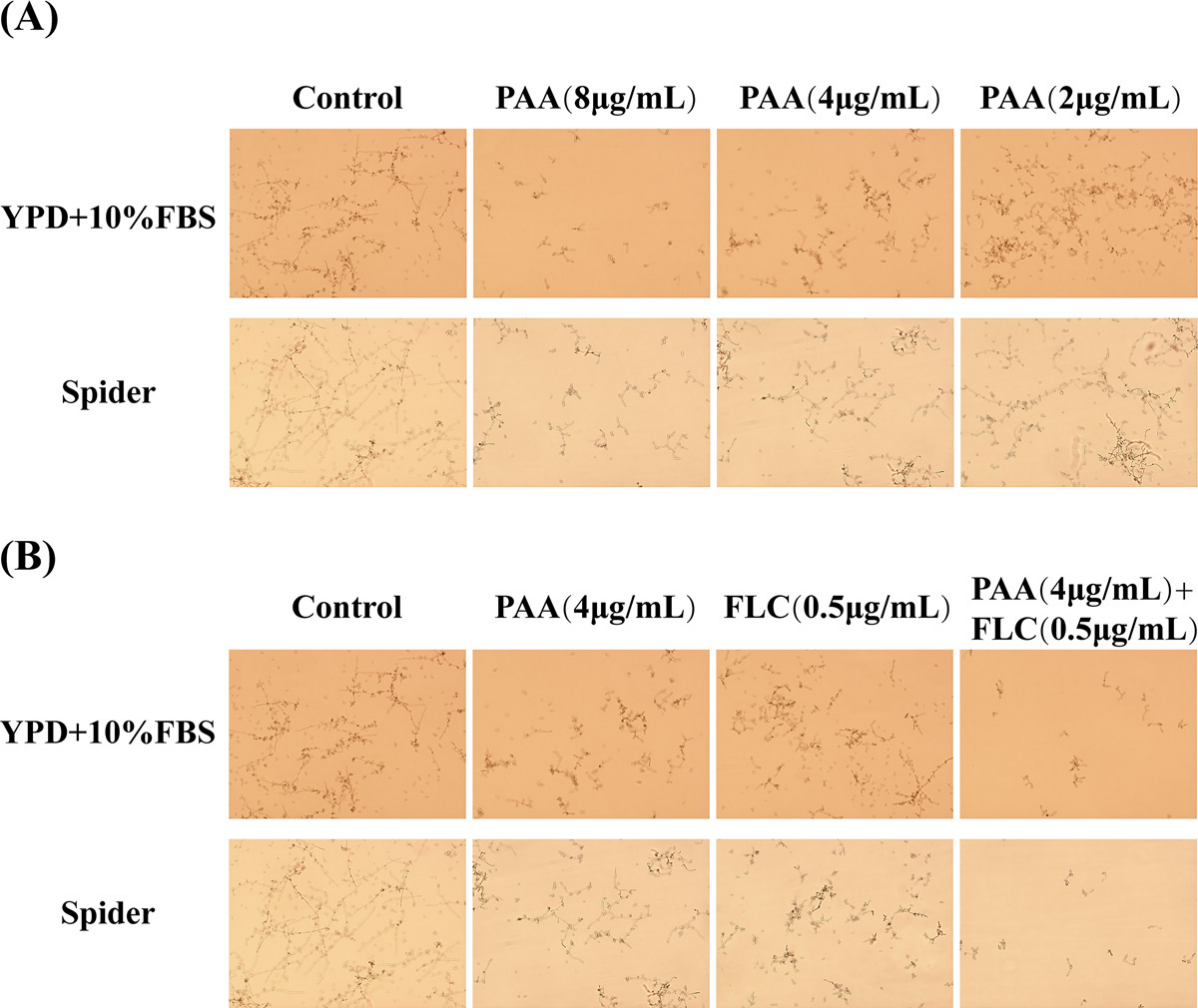
Effect of PAA alone or in combination with FLC on yeast-to-hypha transition of C. albicans during biofilm formation in two different hypha-inducing media. (A) Effect of PAA alone on yeast-to-hypha transition of biofilm formation in spider or RPMI 1640 medium. PAA was diluted in spider or RPMI 1640 medium to final concentrations of 2, 4, and 8 μg/mL. Images were photographed at ×20 magnification. (B) Effect of PAA combined with FLC on yeast-to-hypha transition of biofilm formation in spider or RPMI 1640 medium. C. albicans ATCC 90028 cells were not treated (control; without PAA and FLC) and treated with PAA (4 μg/mL), FLC (0.5 μg/mL), and a combination of PAA (4 μg/mL) and FLC (0.5 μg/mL). Images were photographed at ×100 magnification.

The yeast-to-hypha transition of C. albicans is an important aspect of biofilm development and maintenance (15). C. albicans hyphae induced by spider medium (1% nutrient broth + 1% D-Mannitol +0.2% dipotassium hydrogen phosphate) and yeast extract-peptone-dextrose medium with 10% fetal bovine serum (YPD–10% FBS) are displayed in Fig. 5. As shown, long and extensive hyphae formed a network in both spider medium and YPD–10% FBS. With increasing drug dosages, there was a dose-dependent inhibition effect of PAA on hypha formation in both media (Fig. 5A). Furthermore, when the drug concentration reached 8 μg/mL, PAA shortened the length of hyphae and reduced yeast cell aggregates. The combination PAA-FLC treatment (4-μg/mL PAA+ 0.5-μg/mL FLC) greatly reduced the extent of yeast-to-hypha transition, with the presence of a few dispersed yeast cells. On the contrary, when PAA or FLC was used alone at the same concentration, longish hyphae and dense yeast cell aggregates were observed in the visual field (Fig. 5B). These results suggest that PAA in combination with FLC inhibited yeast-to-hypha transition and hypha formation, thereby inhibiting biofilm formation.

### (iii) PAA combined with FLC altered gene expression during biofilm formation.

**FIG 6:**
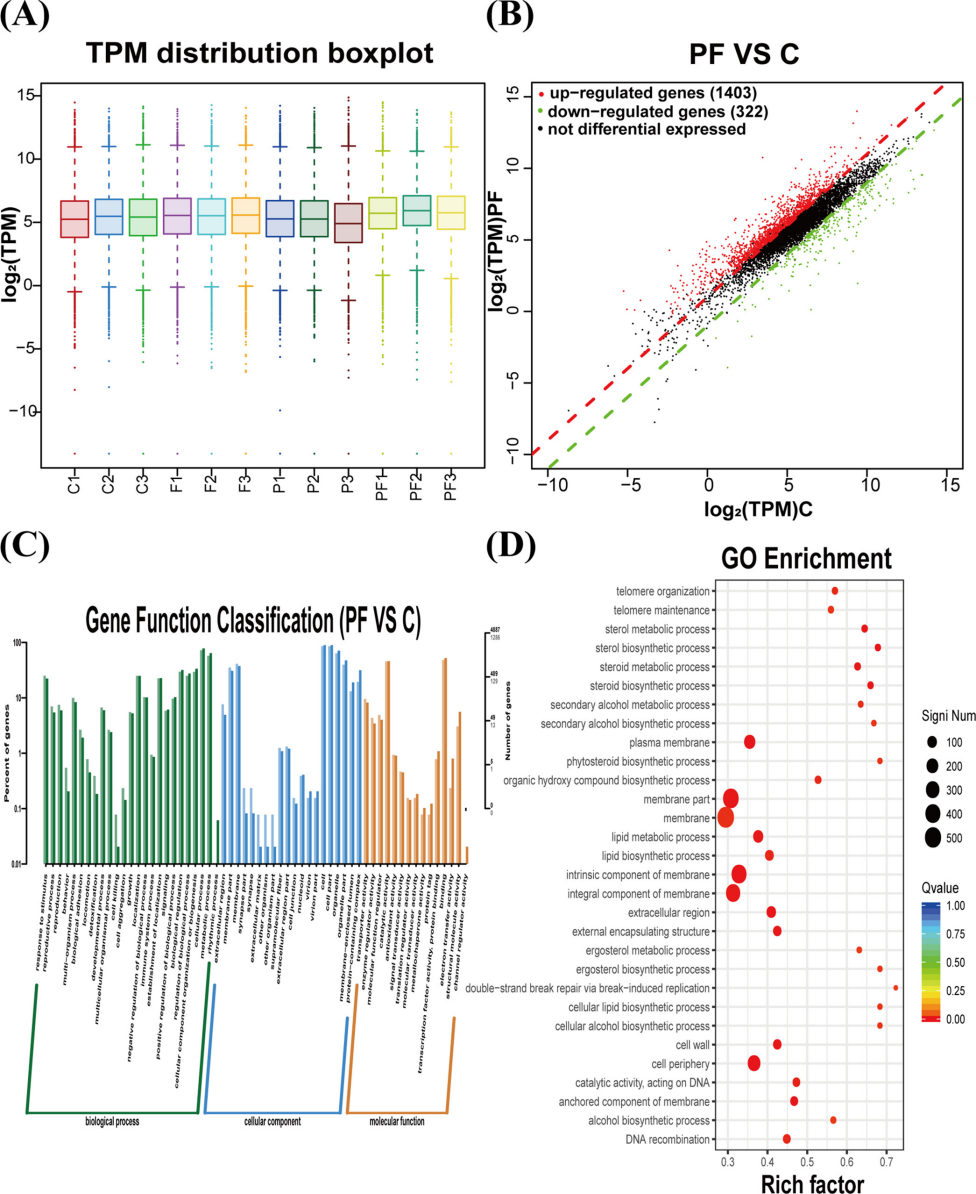
Effect of PAA in combination with FLC on the transcriptome of C. albicans biofilms. (A) The abscissa is the sample name, and the ordinate is the log_2_(TPM) value. TPM, transcripts per million. Box graphs for each region indicate five statistics (from top to bottom, maximum, upper quartile, median, lower quartile, and minimum). (B) The horizontal and vertical coordinates are the log_2_(TPM) values of the PAA-FLC combination group (PF) and control group (C), respectively. (C) The horizontal axis is the functional category, and the vertical axis is the number of genes in the category (right), annotated with its percentage of the total number of genes (left). (D) The vertical axis represents the function annotation information, and the horizontal axis represents the Rich factor corresponding to the function. The Rich factor is the number of differentially expressed genes annotated to that function divided by the number of genes annotated to that function (only the top 30 GO terms with the highest enrichment degree are presented).

The aforementioned experimental results demonstrate that PAA combined with FLC inhibits adhesion and yeast-to-hypha transition in C. albicans. To further investigate the underlying molecular mechanism, gene expression changes were observed under this combination treatment. As shown by the results in Fig. 6A, the total transcriptomes of the four groups (control group, 4-μg/mL PAA group, 0.5-μg/mL FLC group, and 4-μg/mL PAA plus 0.5-μg/mL FLC combination group) were basically consistent. Notably, compared with the control group, a total of 1,725 significantly differentially expressed genes, including 1,403 upregulated and 322 downregulated genes, were observed in the PAA-FLC combination group (*P* < 0.05), as shown in the volcano chart in Fig. 6B The Gene Ontology (GO) classification of genes in the combination treatment is shown in Fig. 6C. The differentially expressed genes were classified into 60 GO categories. Of these, 25 categories were associated with biological processes, 20 categories were associated with cellular components, and 15 categories were associated with molecular functions. Functional enrichment analysis of differentially expressed genes was also performed. Compared with the control group, most of the differentially expressed genes in the PAA-FLC combination group showed different degrees of functional enrichment. The top 30 GO terms with the highest enrichment (*P* < 0.05) were selected (Fig. 6D). Through GO enrichment analysis, the differentially expressed genes were found to be enriched in functions related to double-strand break repair via break-induced replication, sterol biosynthetic process, sterol metabolic process, steroid metabolic process, steroid biosynthetic process, secondary alcohol biosynthetic process, secondary alcohol metabolic process, cellular lipid biosynthetic process, cellular alcohol biosynthetic process, phytosteroid biosynthetic process, ergosterol biosynthetic process, telomere organization, telomere maintenance, alcohol biosynthetic process, and organic hydroxy compound biosynthetic process (Rich factor of >0.5).

**FIG 7:**
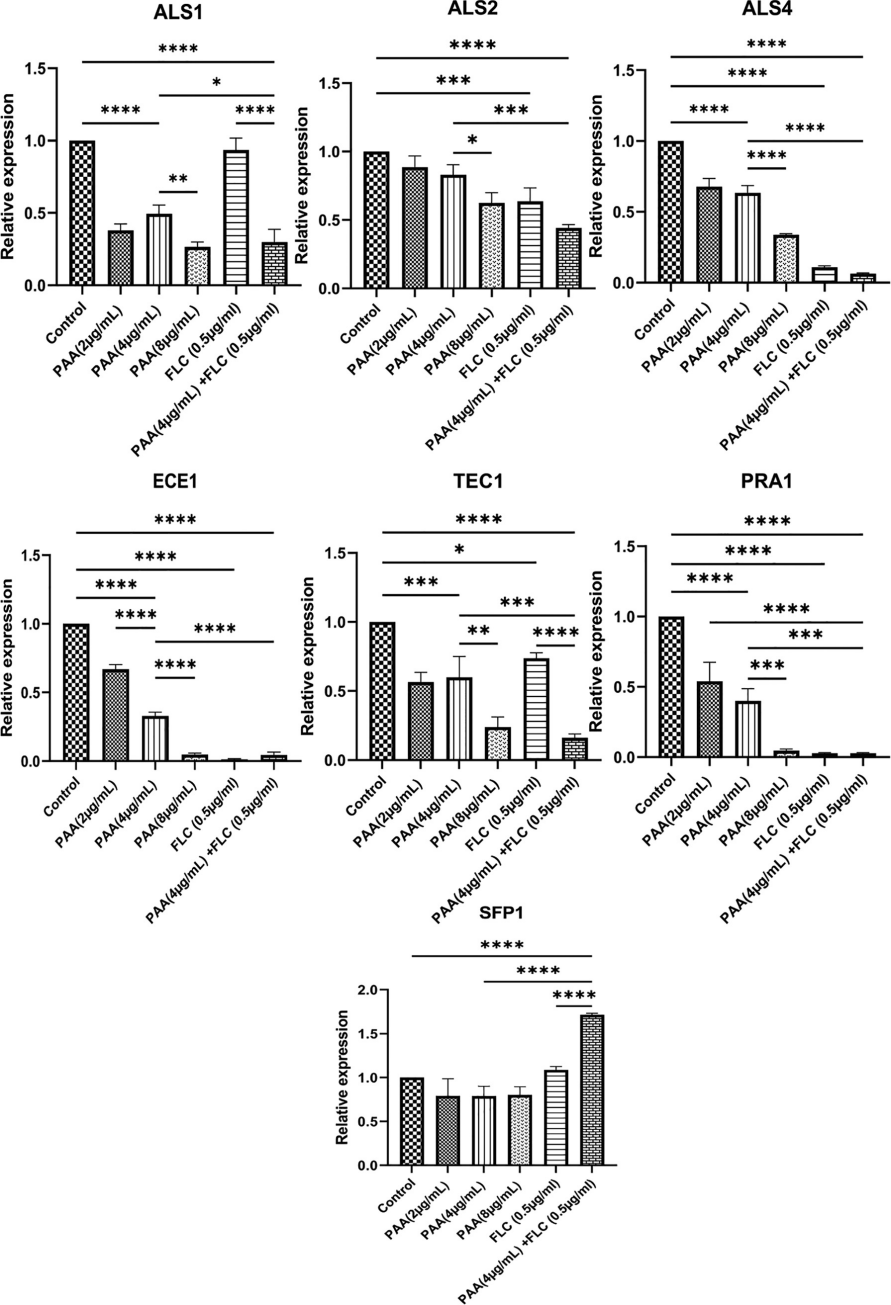
Changes in gene expression related to biofilm formation after PAA and FLC treatment. C. albicans 90028 biofilms were treated with PAA (4 μg/mL) plus FLC (0.5 μg/mL), FLC (0.5 μg/mL), PAA (4 μg/mL), PAA (8 μg/mL), PAA (2 μg/mL), and RPMI 1640 medium (control). Gene expression was measured by qRT-PCR. qRT-PCR was independently repeated three times, with two technical replicates in each experiment. Data represent the mean values ± standard deviations from three triplicate experiments. *P* values were determined by ANOVA. *, *P* < 0.05; **, *P* < 0.01; ***, *P* < 0.001; ****, *P* < 0.0001.

Transcriptome sequencing (RNA-seq) analysis also revealed that the expression levels of genes involved in C. albicans biofilm formation were significantly altered under the combination treatment. To validate the RNA-seq results, four adhesion-associated genes (*ALS1*, *ALS2*, *ALS4*, and *SFP1*) and three hypha-associated genes (*ECE1*, *PRA1*, and *TEC1*) that were found to be related to biofilm formation in the quantitative reverse transcription PCR (qRT-PCR) experiment were selected. The combination treatment significantly altered the expression levels of adhesion-associated and hypha-associated genes, as listed in Table 1. The results presented in Fig. 7 show that PAA (4 μg/ml) treatment significantly downregulated the expression of *ALS4* in comparison to their expression in the control group (*P* < 0.001) (Fig. 7). Increasing PAA concentrations produced more pronounced downregulation of gene expression. Although the expression of ALS4 and ALS2 decreased after FLC treatment, the PAA-FLC combination treatment downregulated the expression of *ALS4* and *ALS2* by 92% and 56% compared to their expression in the control group. Notably, the PAA-FLC combination treatment significantly downregulated the expression of *ALS1* compared with its expression with FLC treatment alone and in the untreated control group (*P* < 0.0001), while the FLC treatment had no significant impact on *ALS1* expression compared with its expression in the control group (*P* > 0.05). *ECE1*, *TEC1*, and *PRA1*, three important yeast-to-hypha transition regulators in C. albicans biofilm formation, were downregulated with the combination treatment in comparison to their expression in the untreated control group (*P* < 0.0001) (Fig. 7). Furthermore, as the concentration of PAA increased, the expression levels of the three genes decreased (*P* < 0.01) (Fig. 7). *SFP1*, the negative regulatory gene of C. albicans biofilm formation, was upregulated after the combination treatment compared with its expression in the control group (*P* < 0.0001) (Fig. 7).

**TABLE 1:** Changes in important gene expression related to adhesion or filamentation in C. albicans biofilms after PAA and FLC treatment compared to the control group determined using RNA-seq

Gene identifier	Gene	Value for indicated treatment in comparison to control^*a*^								
		FLC			PAA-FLC			PAA		
		Log_2_ fold change	*P* value	Significant	Log_2_ fold change	*P* value	Significant	Log_2_ fold change	*P* value	Significant
CAALFM_C603700WA	*ALS1*	0.465	0.356149125	No	−2.005	1.26913E−12	Yes	−0.995	0.024035599	Yes
CAALFM_C604380WA	*ALS2*	−1.894	9.69431E−31	Yes	−1.831	7.89165E−17	Yes	−0.662	0.010456913	Yes
CAALFM_C604130CA	*ALS4*	−2.576	8.71997E−38	Yes	−2.349	9.03704E−19	Yes	−0.681	0.005254679	Yes
CAALFM_C403470CA	*ECE1*	−3.978	1.02707E−07	Yes	−3.657	2.99322E−08	Yes	−1.245	0.109592232	No
CAALFM_C304530CA	*TEC1*	0.678	0.021587397	Yes	−0.916	2.24911E−06	Yes	−0.898	0.001729008	Yes
CAALFM_C406980WA	*PRA1*	−4.359	6.55329E−15	Yes	−3.835	3.44915E−30	Yes	−1.085	0.355181787	No
CAALFM_C304860WA	*SFP1*	0.399	0.079494286	No	1.269	3.65278E−05	Yes	−0.204	0.785353668	No

a Log_2_ fold change values were obtained from RNA-seq results.

## DISCUSSION

Candida albicans is the most common cause of invasive candidiasis worldwide. Strong epidemiological evidence indicates that the mortality of invasive candidiasis is associated with biofilm formation (16, 17). Evidence suggests that C. albicans is more likely to form biofilms than other *Candida* species (such as Candida tropicalis and Candida glabrata) (18). C. albicans biofilms are dynamic and highly structured three-dimensional networks composed of a large number of hyphae and extracellular matrix. These biofilms are resistant to currently available antifungal drugs, especially azoles (19). Compared with planktonic cells, biofilms are up to 1,000 times more resistant to azoles (20). Therefore, in order to decrease the resistance of C. albicans to azole drugs, there is a critical need to identify new effective drugs or treatments that inhibit biofilm formation.

The pine bark of Pseudolarix kaempferi is a traditional Chinese medicine that has long been used to treat the dermatomycosis caused by microbe infection (21). Pseudolaric acid A (PAA) is a diterpenoid mainly derived from the pine bark of Pseudolarix kaempferi; it has a certain inhibitory effect on C. albicans. However, few published studies have investigated the effect of PAA alone or in combination with fluconazole (FLC) against C. albicans biofilm formation. In this study, we evaluated the inhibitory effect of PAA alone or in combination with FLC against C. albicans biofilm formation *in vitro*.

The formation of C. albicans biofilms involves several specific stages, including the early, developmental, and mature phases (14). In the present study, we observed a significant synergistic effect of PAA in combination with FLC against C. albicans biofilm formation. Changes in the fluorescence intensity of the fluorescein diacetate (FDA) assay further confirmed that PAA in combination with FLC significantly decreased the metabolic activity and growth of biofilm cells. The antibiofilm activity of PAA-FLC was stronger than that of PAA or FLC alone. Interestingly, the antibiofilm effect was mainly related to the dose of PAA in the mature biofilm phase, while the antibiofilm effect was primarily correlated with the concentration of FLC in the early and developmental phases of biofilm formation. We hypothesize that the reason for this may be that the synergistic antibiofilm effect of PAA and FLC probably involves different antifungal targets. Terpenoids can lead to membrane damage and respiratory chain dysfunction in C. albicans cells, which affects biofilm formation (22). The specific antibiofilm mechanism remains to be further studied, although PAA is a diterpenoid.

Cell adherence is the first step in biofilm formation. After the adhesion of C. albicans cells, the yeast cells gradually transform into hyphal forms, which is important not only for biofilm development but also for biofilm dissemination (23). Adhesion and yeast-to-hypha transition are considered the crucial pathogenic elements. Inhibition of adhesion or yeast-to-hypha transition can induce biofilm formation defects, which is a novel target for biofilm-specific treatment (24, 25). Our data showed that the PAA-FLC combination treatment not only inhibited adhesion effectively but also suppressed yeast-to-hypha transition. The inhibition of adhesion and yeast-to-hypha transition in the combination treatment (PAA-FLC) was stronger than that produced by the individual drugs. The synergistic antibiofilm action of the PAA-FLC combination was likely due to inhibition of adhesion and yeast-to-hypha transition.

To investigate the synergistic antibiofilm mechanisms of PAA combined with FLC, RNA-seq and qRT-PCR were used to analyze changes at the transcriptional and genetic levels. Both independent trials indicated that the combined use of PAA and FLC effectively altered the expression of *ALS1*, *ALS4*, *ALS2*, *SFP1*, *ECE1*, *TEC1*, and *PRA1*. The *ALS* family, including *ALS1*, *ALS2*, and *ALS4*, contributes to the adhesion and aggregation of yeast cells and is an integral part of biofilm formation (26). *SFP1* is one of the few negative regulators of adhesion, and the deletion of *SFP1* results in the promotion of biofilm formation (27). *ECE1* is a hyphal induction gene that stimulates hypha formation and elongation (28). *TEC1* and *PRA1*, the master regulatory genes involved in biofilm formation, play crucial roles in hyphal development (29, 30). Notably, *TEC1* is important for regulating the morphological switch between yeast and hypha (31). The aforementioned phenotypic results indicate that the PAA-FLC combination had a strong effect on the inhibition of adhesion and yeast-to-hypha transition during biofilm formation. The PAA-FLC combination treatment downregulated the expression of adhesion-related genes (*ALS4*, *ALS2*, and *ALS1*) and hypha-related genes (*ECE1*, *TEC1*, and *PRA1*) during biofilm formation. At the same time, the PAA-FLC combination treatment remarkably increased the expression of the negative-regulation gene (*SFP1*). The genotypic and phenotypic results were correlated. PAA and FLC may have various antibiofilm targets, which contributes to their synergistic effect. Both PAA and FLC alone had certain inhibitory effects on the gene expression of *ALS2*, *ALS4*, *ECE1*, and *PRA1*. However, PAA in combination with FLC produced more significant inhibitory effects on these genes compared to either of the drugs alone. Notably, PAA, rather than FLC, may have a primary role in suppressing the expression of *ALS1*. These findings reveal the deep mechanism underlying the synergistic inhibition of C. albicans biofilm formation by PAA in combination with FLC.

In conclusion, the present study demonstrates, for the first time, the antibiofilm effect of PAA alone and in combination with FLC on C. albicans. PAA exhibited better synergistic inhibitory effects on C. albicans biofilm formation when combined with FLC. Furthermore, PAA in combination with FLC inhibited adhesion and hyphal morphogenesis during biofilm formation. Moreover, PAA-FLC combination treatment not only specifically inhibited adhesion- and hypha-related genes but also increased the expression of the gene that negatively regulates biofilm formation. These results provide novel insights into the synergistic effect and mechanisms of PAA, a traditional Chinese medicine, combined with antifungal drugs.

## MATERIALS AND METHODS

### Strains, growth conditions, and reagents.

The reference strain C. albicans ATCC 90028 was used in all experiments. C. albicans ATCC 90028 was preserved in a medium containing 20% glycerol at −80°C. The strain was subcultured twice on Sabouraud agar plate medium (Comagal Microbial Technology Co., Shanghai, China) for 16 h at 37°C before each experiment. PAA was obtained from Tauto Biotech Co., Ltd. (Shanghai, China). FLC with a purity of >98% was purchased from Sigma-Aldrich. Both PAA and FLC were dissolved in dimethyl sulfoxide (DMSO); the final concentration of DMSO was lower than 0.1%. In addition, RPMI 1640 was obtained from Fisher Scientific. MOPS [3-(*N*-morpholino) propanesulfonic acid] was obtained from Sangon Biotech, Shanghai. Tetrazolium {XTT [2,3-bis-(2-methoxy-4-nitro-5-sulfophenyl)-2H-tetrazolium-5-carboxanilide salt]} and fluorescein diacetate were purchased from Sigma-Aldrich.

### Antibiofilm formation. (i) XTT reduction assay.

The effects on biofilm formation of PAA or FLC alone and in combination were determined by XTT reduction assays (32, 33). Biofilms were generated on commercial polystyrene 6-well microtiter plates. In brief, 100 μL of C. albicans ATCC 90028 (about 1.5 × 10^6^ CFU/mL) in RPMI 1640 medium was seeded into 96-well plates for biofilm formation. At different phases of biofilm formation (1.5, 6, or 24 h), the medium and planktonic cells in each well were removed and washed with phosphate-buffered saline (PBS). Then, 100 μL of RPMI 1640 medium containing PAA or FLC was added to each well. In terms of the antibiofilm activity of the combination treatment, 50 μL of RPMI 1640 medium containing PAA alone (or FLC) was placed in each well at planned time points based on the microdilution chequerboard approach. After the plates were incubated at 37°C for 24 h, the biofilm in each well was washed gently three times with 100 μl PBS to remove the planktonic cells. A cell proliferation kit II (XTT; Sigma-Aldrich) was used to evaluate the effect of biofilm formation. XTT and electron coupling reagent were mixed at 50:1 (vol/vol) immediately prior to the assay. Afterwards, PBS was added to the XTT mixture (1.96:1, vol/vol) and 151 μl of this mixture was placed into each well. Then, the plates were incubated in the dark for 2 h at 37°C. The colored supernatants were transferred to new 96-well plates, and the optical density at 492 nm (OD_492_) was measured. Each experiment was performed in triplicate.

### (ii) FDA assay.

A fluorescein diacetate (FDA) assay was used to quantify the viable biofilm biomass (34, 35). In brief, C. albicans ATCC 90028 (about 1.5 × 10^6^ CFU/mL) in RPMI 1640 medium was transferred into 6-well plates for biofilm formation. After incubation (1.5, 6, or 24 h), planktonic cells were removed, and the biofilms were left untreated or were treated with 4 μg/mL PAA, 0.5 μg/mL FLC, or 4 μg/mL PAA in combination with 0.5 μg/mL FLC for 24 h at 37°C. Each well was washed with PBS three times to remove the planktonic cells. Next, 0.2 mg/mL of FDA (Sigma-Aldrich, Shanghai, China) and fresh RPMI 1640 medium were mixed 1:1 (vol/vol) immediately prior to the assay. The FDA mixture was then transferred into each prerinsed well. The 6-well plates were incubated at 37°C for 1 h in the dark. Before observation, the plates were gently rinsed with PBS two times to remove the dye residue. The morphology of the biofilms was observed under a fluorescence microscope (EUROStar III plus; Ou Meng, Germany) at ×200 magnification.

### (iii) Cell adhesion assay.

The *in vitro* cell adherence assay was performed according to previously reported methods, with some modifications (36, 37). An overnight culture of C. albicans ATCC 90028 was adjusted in RPMI 1640 medium to a final concentration of about 1.5 × 10^6^ CFU/mL. Amounts of 200 μL of cell suspensions were inoculated into the wells of a 96-well plate and PAA (4 μg/mL), FLC (0.5 μg/mL), or the combination of the two was added to the cell suspensions. C. albicans cell suspensions without medication were regarded as the control. After the plates were incubated at 37°C for 3 h, the liquid medium was thrown away. Each well containing cells was washed three times. Then, the XTT mixture with PBS, as described above, was added to each well, and the cells were incubated in the dark for 2 h at 37°C. The supernatants obtained were transferred to a new 96-well plate and the OD_492_ was analyzed. The relative adhesion rate of C. albicans cells was expressed as follows: adhesion rate = (OD_492_ of treatment/OD_492_ of negative control) × 100 % (38). Each experiment was carried out in triplicate.

### Yeast-to-hypha transition assay.

The effect of PAA alone and in combination with FLC on the yeast-to-hypha phase transition of C. albicans was measured according to previously described methods, with minor alterations (39, 40). C. albicans ATCC 90028 was incubated overnight in YPD medium. Then, the fungal supernatant (about 1.5 × 10^5^ CFU/mL) with PAA (at 4 μg/mL), FLC (0.5 μg/mL), or the combination of the two was incubated in YPD–10% FBS or spider medium at 37°C for 6 h. The fungal supernatant without drug treatment served as the control. Samples were incubated under aerobiotic conditions with continuous shaking at 120 rpm. After 6 h of incubation, 20 μL of cell suspension was transferred to a fast counter plate to observe the yeast cells and hyphae form under a light microscope with a ×10 magnification eyepiece and 10× lens objective.

### RNA-seq analysis of C. albicans biofilms.

First, 1.5 mL of C. albicans ATCC 90028 (about 1.5 × 10^6^ CFU/mL) in RPMI 1640 medium was seeded into 6-well polystyrene plates and cultured at 37°C for 1.5 h. After 1.5 h of incubation for biofilm formation, 1.5 mL of RPMI 1640 medium containing PAA (4 μg/mL), FLC (0.5 μg/mL), or the combination of the two was added to each well, and the plates were incubated for a further 24 h at 37°C. The control wells were free of FLC and PAA. Then, the biofilms were gently washed three times with PBS and collected with a sterile cell scraper. Total RNA from the C. albicans biofilms was extracted by using a nucleic acid purification kit (MagExtractor; Toyobo Co., Ltd., Osaka, Japan) following the manufacturer’s instructions; genomic DNA contamination was removed using RNase-free DNase I (Toyobo). The quality and quantity of RNA samples were checked using a Qubit RNA assay kit (Life Technologies, CA, USA) before RNA sequencing. High-quality RNA samples were sent to Sangon Biotech company, Shanghai, China, for library preparation and sequencing. The sequencing libraries were prepared using the VAHTS mRNA-seq version 2 library prep kit for Illumina according to the manufacturer’s protocols, and sequencing was performed on a NovaSeq 6000 sequencer (Illumina, Inc., USA). The quality of the RNA sequencing data was analyzed using FastQC (http://www.bioinformatics.babraham.ac.uk/projects/fastqc/). The raw data were quality filtered using Trimmomatic (TRAILING: 30) to obtain relatively accurate and clean data. HISAT2 (version 2.0.1) was used to map the clean reads to the reference genome and the alignment results were counted using RSeQC (version 2.6.1). The expression of genes was quantified using StringTie (version 1.3.3), and differential analysis of gene expression was carried out using DESeq2 (version 1.20.0). Gene Ontology (GO) enrichment analysis was carried out using the topGO R package in order to acquire the gene functions of differentially expressed genes. The experiments were repeated in triplicate with independent trials.

### Specific gene expression related to biofilm formation by qRT-PCR.

C. albicans suspensions (ATCC 90028) with a cell density of about 1.5 × 10^6^ CFU/mL were seeded into 6-well polystyrene plates and cultured at 37°C for 1.5 h. After 1.5 h of incubation, PAA (2 μg/mL), PAA (4 μg/mL), PAA (8 μg/mL), FLC (0.5 μg/mL), or PAA (4 μg/mL) plus FLC (0.5 μg/mL) was added, and the plates were incubated for a further 24 h at 37°C. The biofilms were gently washed three times with PBS and collected with a sterile cell scraper. Total RNA from the C. albicans biofilms was extracted with a nucleic acid purification kit (MagExtractor, Toyobo Co., Ltd., Osaka, Japan) according to the manufacturer’s instructions. Then, about 1 μg of total RNA was treated with a RevertAid first-strand cDNA synthesis kit (Thermo Scientific, Inc.) and was reverse transcribed at 42°C for 1 h and 70°C for 5 min. qRT-PCRs were carried out using SYBR green real-time PCR master mix (Toyobo Co., Ltd., Osaka, Japan) on a Bio-Rad CFX96 real-time system (Bio-Rad). Each reaction mixture volume (25 μL) contained 12.5 μL of SYBR green master mix, 1 μL of cDNA, 1 μL of forward primer, 1 μL of reverse primer, and 9.5 μL of distilled water. Primers for C. albicans biofilms were designed and synthesized by BioSune Biotechnology Co., Ltd. (Shanghai, China). The primer sequences used to amplify gene segments are shown in Table 2. For qRT-PCR, the thermal cycling conditions were as follows: 95°C for 60 s, followed by 40 cycles at 95°C for 10 s, annealing at 60°C for 15 s, and extension at 72°C for 45 s. After qRT-PCR, melting curve analysis was carried out to check for nonspecific amplification. The target gene expression was calculated by the 2^–ΔΔ^*^CT^* cycle threshold (*C_T_*) method using the *ACT* gene as the internal reference gene; the Δ*C_T_* value was expressed as the average *C_T_* value for the target gene minus the average *C_T_* value for *ACT*. qRT-PCR assays for each gene were performed in triplicate.

**TABLE 2:** Primer sequences used for qRT-PCR in the study

Primer	Sequence (5′–3′)	Length (bp)	Reference or source
ACT1-Forward	TCAGACCAGCTGATTTAGGTTTG	23	41
ACT1-Reverse	GTGAACAATGGATGGACCAG	20	41
ALS1-Forward	TTGGGTTGGTCCTTAGATGG	20	42
ALS1-Reverse	ATGATTCAAAGCGTCGTTC	19	42
ALS2-Forward	TGGTGCAATGGGGTTCATAGT	21	43
ALS2-Reverse	CGATAACCAGCGGGGACAT	19	43
ALS4-Forward	TCCGAGTCCATTCCAGTACTAA	22	44
ALS4-Reverse	GTTACAGCATCACTAGAAGGAATATC	26	44
ECE1-Forward	GCTGGTATCATTGCTGATAT	20	42
ECE1-Reverse	TTCGATGGATTGTTGAACAC	20	42
TEC1-Forward	TCAACAGTCACGAGGAA	17	45
TEC1-Reverse	TGGCTGGGAGATGC	14	45
PRA1-Forward	GGCGAAGGGTGCAATGGAGATG	22	This study
PRA1-Reverse	GAGAACTTGAGGCTGTGCTACTGG	24	This study
SFP1-Forward	GCACGTCACCTCTACGTTATGG	22	This study
SFP1-Reverse	CGATGACCGGGCACTTG	17	This study

### Statistical analysis.

Graphs and statistical analyses were performed with GraphPad Prism 9 (GraphPad software, Inc.). Differences among three or more groups were evaluated by analysis of variance (ANOVA), while intergroup differences were further determined using the Bonferroni method. *P* values less than 0.05 (*, *P* < 0.05, **, *P* < 0.01, ***, *P* < 0.001, ****, *P* < 0.0001) were considered as statistically significant.

### Data availability.

The RNA-seq data from this study have been deposited in the NCBI database under the BioProject accession number PRJNA757949.
