# Supplementary material for: Synergistic Antibiofilm Effects of Pseudolaric Acid A Combined with Fluconazole against Candida albicans via Inhibition of Adhesion and Yeast-To-Hypha Transition
Source: Microbiol Spectr. 2022 Mar 17;10(2):e01478-21. doi: 10.1128/spectrum.01478-21 (PMC9045105; doi:10.1128/spectrum.01478-21)
Supplement: SUPPLEMENTAL FILE 1 — Supplemental material. Download SPECTRUM01478-21_Supp_1_seq2.pdf, PDF file, 0.2 MB [file spectrum01478-21_supp_1_seq2.pdf]

**Suppl Table S1 | ** List of common differentially expressed genes of *C. albicans* biofilm by PAA or FLC or PAA in combination with FLC

C. albicans biofilm was treated with control (without PAA and FLC), PAA(4μg/mL), FLC(0.5μg/mL), and a combination of PAA(4μg/mL) with FLC(0.5μg/mL). RNA-seq assays were used to determine differential gene expression.

Gene id	Gene Name	FLC vs Control			PAA+FLC vs Control			PAA vs Control		
		log2FoldChange	P value	Significant	log2FoldChange	P value	Significant	log2FoldChange	P value	Significant
CAALFM_C603700WA	ALS1	0.465	0.356149125	no	-2.005	1.26913E-12	yes	-0.995	0.024035599	yes
CAALFM_C604380WA	ALS2	-1.894	9.69E-31	yes	-1.831	7.89165E-17	yes	-0.662	0.010456913	yes
CAALFM_C604130CA	ALS4	-2.576	8.72E-38	yes	-2.349	9.04E-19	yes	-0.681	0.005254679	yes
CAALFM_C403570WA	HWP1	-2.926	3.41885E-55	yes	-1.063	9.02095E-06	yes	-0.954	0.00019936	yes
CAALFM_C403510CA	HWP2	1.427	0.008579198	yes	1.928	0.004302732	yes	0.726	0.052910335	no
CAALFM_C304530CA	TEC1	0.678	0.021587397	yes	-0.916	2.24911E-06	yes	-0.898	0.001729008	yes
CAALFM_C403470CA	ECE1	-3.978	1.02707E-07	yes	-3.657	2.99322E-08	yes	-1.245	0.109592232	no
CAALFM_C210210CA	RAS1	-1.417	1.36784E-18	yes	-1.035	7.12886E-10	yes	-0.164	0.896298286	no
CAALFM_C304480CA	RAS2	1.959	1.06706E-13	yes	1.904	4.39711E-10	yes	0.280	0.057900468	no
CAALFM_C304860WA	SFP1	0.399	0.079494286	no	1.269	3.65278E-05	yes	-0.204	0.785353668	no
CAALFM_C600820WA	SUN41	-1.345	1.48577E-15	yes	0.775	0.177512838	no	-0.192	0.826688345	no
CAALFM_C702030WA	HSP90	-1.997	5.91106E-28	yes	-3.679	3.04522E-63	yes	0.096	0.240049142	no
CAALFM_C406980WA	PRA1	-4.359	6.55329E-15	yes	-3.835	3.44915E-30	yes	-1.085	0.355181787	no
CAALFM_C603850CA	IHD1	-4.996	1.79946E-70	yes	-1.891	2.77903E-11	yes	-1.188	0.008456303	yes
CAALFM_C106280CA	UME6	-0.675	0.084385865	yes	3.272	0.000168511	yes	-0.748	0.201454255	no
CAALFM_C600470CA	FET99	4.013	3.97935E-21	yes	1.855	0.00000426	yes	3.616	5.45189E-14	yes
CAALFM_C106260WA	PGA50	-3.934	0.002207634	yes	0.459	0.380855058	no	-0.162	0.504567306	no
CAALFM_C105960WA	PGA45	3.421	9.76227E-34	yes	2.946	5.31404E-17	yes	0.735	0.006551277	yes
CAALFM_C203220CA	STP4	2.824	2.18179E-69	yes	3.048	1.12113E-22	yes	0.185	0.294797151	no
CAALFM_C402030WA	Rfx2	1.219	2.32773E-10	yes	0.069	0.22406432	no	2.540	9.3245E-20	yes

CAALFM_C114280CA	Npl3	-1.382	5.28522E-17	yes	-1.269	1.2511E-11	yes	-0.338	0.33532389	no
CAALFM_C502840CA	HSL1	-0.999	1.68361E-12	yes	1.314	0.001873652	yes	-0.218	0.755127106	no
CAALFM_C704230WA	NRG1	1.342	1.56021E-09	yes	0.965	0.01118957	yes	-0.318	0.539166152	no
CAALFM_C601140CA	ARC18	0.616	0.098865252	no	1.382	0.000965189	yes	0.076	0.378442589	no
CAALFM_C108460CA	UPC2	2.637	1.34357E-26	yes	2.582	6.49467E-14	yes	-0.130	0.834179522	no
CAALFM_C100780CA	HGC1	1.954	3.37771E-11	yes	1.700	1.13989E-05	yes	0.264	0.149665976	no
CAALFM_C505050WA	ADH1	-0.817	1.40793E-05	yes	-1.520	1.44489E-11	yes	0.242	0.166313488	no
CAALFM_C210290WA	SLR1	-0.498	0.003778626	yes	-1.740	2.29355E-20	yes	0.193	0.095327872	no
CAALFM_C104240CA	HHF1	-2.217	9.35039E-21	yes	-1.842	8.61378E-16	yes	-0.569	0.182325843	no
CAALFM_C100220WA	PHR2	2.208	3.74931E-26	yes	3.077	9.92068E-27	yes	-0.298	0.542393079	no
CAALFM_CR06860CA	ARO10	-1.317	1.70717E-08	yes	-1.035	9.22484E-08	yes	-0.303	0.93384098	no
CAALFM_C305620WA	ATO5	0.772	0.037968384	yes	1.594	0.000119678	yes	0.917	5.79625E-05	yes
CAALFM_CR00740CA	BMT3	1.371	8.90245E-14	yes	2.015	1.30893E-12	yes	0.096	0.213343061	no
CAALFM_C304890WA	CDR2	1.187	8.4501E-06	yes	2.236	6.76042E-11	yes	-0.518	0.157912364	no
CAALFM_C504130CA	CHT2	-2.606	1.63233E-35	yes	-2.329	8.5859E-26	yes	-0.624	0.084636559	no
CAALFM_C109250WA	CRP1	-1.395	1.94279E-09	yes	-2.336	7.29409E-28	yes	0.366	0.072182651	no
CAALFM_CR09880WA	DEF1	0.902	3.35437E-05	yes	1.506	2.35494E-05	yes	-0.530	0.058386946	no
CAALFM_C114220CA	FTR2	-2.448	3.06312E-17	yes	-1.630	0.001035333	yes	-0.393	0.416830689	no
CAALFM_C106610CA	HAK1	2.952	7.92169E-33	yes	2.353	1.21682E-11	yes	0.642	0.000553352	yes
CAALFM_C503310CA	PCL1	0.133	0.951714585	no	2.043	0.000128537	yes	-0.233	0.717527897	no
CAALFM_C103820WA	PDR16	0.560	0.003977326	yes	2.111	5.75288E-12	yes	-0.265	0.647740334	no
CAALFM_C404080CA	PGA31	0.911	0.297353318	no	3.010	0.000670305	yes	-0.976	0.063117621	no
CAALFM_C502240WA	PTH2	1.308	7.33385E-06	yes	0.922	0.06806787	no	-0.180	0.926862592	no
CAALFM_C105000WA	RHD1	2.564	0.000852971	yes	1.928	0.041809905	yes	0.015	0.760098892	no

CAALFM_C404050CA	RHD3	-2.551	1.21421E-24	yes	0.630	0.800150341	no	0.659	0.002021236	yes
CAALFM_C206460WA	RTA3	1.867	4.60569E-12	yes	2.416	8.63454E-17	yes	-0.233	0.732369454	no
CAALFM_C503060CA	TNA1	-2.226	0.000269755	yes	-2.865	5.03685E-18	yes	-0.014	0.918346262	no
CAALFM_C305050WA	TRY4	-1.288	6.68699E-20	yes	-1.610	1.2923E-24	yes	0.224	0.056715594	no
CAALFM_C209990CA	YOR1	0.548	0.055291812	no	1.435	1.99304E-07	yes	-0.155	0.910982629	no

Suppl Table S2 | List of down-regulation genes in the top 50 in terms of expression difference multiples (log2FoldChange of the PAA+FLC vs Control, P<0.05) *C. albicans* biofilm was treated with control (without PAA and FLC), PAA(4μg/mL), FLC(0.5μg/mL), and a combination of PAA(4μg/mL) with FLC(0.5μg/mL). RNA-seq assays were used to determine differential gene expression.

Gene id	Gene Name	FLC vs Control			PAA+FLC vs Control			PAA vs Control		
		log2FoldChange	P value	Significant	log2FoldChange	P value	Significant	log2FoldChange	P value	Significant
CAALFM_C102540CA	tR(UCU)4	-2.945	0.000116714	yes	-7.453	1.99547E-59	yes	-0.028	0.823942756	no
CAALFM_C204890CA	tD(GUC)5	-4.880	6.48201E-16	yes	-6.361	4.06111E-21	yes	0.364	0.59460205	no
CAALFM_CR02320WA	hypothetical protein	-1.478	0.183333206	no	-5.172	0.007864274	yes	-0.086	0.988251327	no
CAALFM_C204000CA	hypothetical protein	-3.000	0.130239388	no	-4.911	0.008011375	yes	-7.416	4.11934E-06	yes
CAALFM_C101990WA	HSP30	-5.003	1.0067E-98	yes	-4.866	6.94965E-54	yes	1.149	0.04156586	yes
CAALFM_C303350WA	tE(UUC)1	-3.069	4.01873E-09	yes	-4.607	1.0273E-18	yes	0.699	0.326885891	no
CAALFM_C305500WA	SNR6	-1.123	0.08458554	no	-4.495	7.1425E-05	yes	-0.778	0.409449256	no
CAALFM_CR08250CA	HSP104	-2.618	1.5803E-47	yes	-4.400	8.8108E-114	yes	-0.292	0.513003166	no
CAALFM_CR08270WA	hypothetical protein	-2.319	3.32119E-17	yes	-4.311	1.45342E-94	yes	-0.575	0.159992397	no
CAALFM_C104300CA	SSA2	-2.482	7.25385E-42	yes	-4.295	8.43078E-96	yes	-0.216	0.817477283	no

CAALFM_C203390CA	HSP78	-2.405	7.98116E-44	yes	-4.090	3.3507E-100	yes	0.239	0.050727218	no
CAALFM_C300920WA	ATO1	-5.444	1.82818E-65	yes	-4.027	3.30094E-07	yes	-0.994	0.011179343	yes
CAALFM_C113480WA	HSP70	-2.374	5.2423E-54	yes	-3.921	1.4777E-102	yes	-0.004	0.384824304	no
CAALFM_C204010CA	HSP21	-2.573	1.56888E-68	yes	-3.866	4.8257E-35	yes	0.391	0.023336101	yes
CAALFM_C406980WA	PRA1	-4.359	6.55329E-15	yes	-3.835	3.44915E-30	yes	-1.085	0.355181787	no
CAALFM_C401940WA	PHO89	-4.645	1.83047E-47	yes	-3.809	2.38146E-37	yes	-0.680	0.105117314	no
CAALFM_C111320CA	hypothetical protein	-3.825	1.43313E-53	yes	-3.778	1.81963E-67	yes	0.474	0.00169074	yes
CAALFM_CR06810WA	HHT2	-4.308	7.95811E-45	yes	-3.756	5.76558E-39	yes	-1.038	0.032706117	yes
CAALFM_C702030WA	HSP90	-1.997	5.91106E-28	yes	-3.679	3.04522E-63	yes	0.096	0.240049142	no
CAALFM_C403470CA	ECE1	-3.978	1.02707E-07	yes	-3.657	2.99322E-08	yes	-1.245	0.109592232	no
CAALFM_C406970CA	ZRT1	-4.141	5.58473E-14	yes	-3.523	2.25647E-27	yes	-0.678	0.53729716	no
CAALFM_CR10790WA	MAL2	-3.324	3.14385E-51	yes	-3.480	2.18506E-55	yes	0.761	0.000161587	yes
CAALFM_C204940CA	ITR1	-1.581	2.18829E-16	yes	-3.421	8.92917E-61	yes	-0.051	0.577395141	no
CAALFM_CR06490CA	HSP60	-1.894	7.49057E-21	yes	-3.407	8.00625E-54	yes	0.141	0.188897443	no
CAALFM_C104260WA	HHT21	-4.061	7.45997E-40	yes	-3.400	1.20428E-33	yes	-0.917	0.073882188	no
CAALFM_C501820WA	STI1	-1.859	1.02787E-23	yes	-3.389	2.25343E-60	yes	0.142	0.163574405	no
CAALFM_C504980WA	hypothetical protein	-2.201	7.10551E-17	yes	-3.363	1.54566E-42	yes	1.093	5.03568E-05	yes
CAALFM_CR10270CA	AHA1	-2.014	5.89159E-32	yes	-3.296	5.2777E-60	yes	-0.098	0.720400488	no
CAALFM_C601860CA	HCH1	-2.162	5.07088E-24	yes	-3.211	1.82469E-50	yes	0.062	0.34663038	no
CAALFM_CR08260CA	hypothetical protein	-2.118	2.11711E-20	yes	-3.207	2.19635E-56	yes	-0.168	0.924762027	no
CAALFM_C404020CA	hypothetical protein	-4.142	4.46654E-68	yes	-3.108	2.56595E-07	yes	-0.504	0.142852427	no

CAALFM_C504210CA	MRV4	-3.476	3.14393E-29	yes	-3.036	1.62073E-37	yes	0.608	0.013967797	yes
CAALFM_C110170WA	hypothetical protein	-2.845	1.18027E-29	yes	-2.972	5.31828E-29	yes	0.898	0.010254095	yes
CAALFM_CR00300WA	CDA2	-3.259	6.49833E-40	yes	-2.906	3.88891E-41	yes	0.577	0.214544625	no
CAALFM_C700350CA	hypothetical protein	-1.964	3.1638E-33	yes	-2.888	1.30364E-64	yes	-0.027	0.401713633	no
CAALFM_C503060CA	TNA1	-2.226	0.000269755	yes	-2.865	5.03685E-18	yes	-0.014	0.918346262	no
CAALFM_C200860CA	hypothetical protein	-2.413	1.22358E-12	yes	-2.858	3.42872E-21	yes	-0.599	0.695127418	no
CAALFM_C504930CA	MAL31	-4.238	5.86922E-42	yes	-2.819	7.86563E-24	yes	-0.213	0.996195116	no
CAALFM_C114090WA	hypothetical protein	-1.452	6.77093E-14	yes	-2.796	3.65901E-39	yes	0.407	0.013998377	yes
CAALFM_C209820WA	hypothetical protein	-1.298	1.78434E-09	yes	-2.782	1.54917E-27	yes	0.144	0.237191198	no
CAALFM_C503600WA	MDJ1	-1.400	4.44573E-10	yes	-2.754	1.13989E-32	yes	0.249	0.127504579	no
CAALFM_C202590WA	ZRT2	-1.999	3.17912E-12	yes	-2.733	9.33928E-24	yes	-0.406	0.79172137	no
CAALFM_CR02750CA	PGA34	-5.530	2.11393E-18	yes	-2.672	8.35083E-13	yes	-0.464	0.342752517	no
CAALFM_C203640WA	UGA11	-1.059	0.000212765	yes	-2.663	2.86298E-21	yes	-0.067	0.830501014	no
CAALFM_C113100WA	hypothetical protein	-0.220	0.234835405	no	-2.656	8.5302E-27	yes	-0.222	0.761783272	no
CAALFM_C600290WA	hypothetical protein	-0.444	0.0427232	yes	-2.647	5.71404E-22	yes	0.327	0.106220848	no
CAALFM_CR02490WA	OPT4	-3.291	1.55284E-08	yes	-2.634	1.87809E-06	yes	-0.430	0.67118868	no
CAALFM_CR06800CA	HHF22	-3.332	2.20342E-38	yes	-2.605	1.76428E-21	yes	-0.715	0.101402169	no

Suppl Table S3 | List of up-regulation genes in the top 50 in terms of expression difference multiples ((log2FoldChange of the PAA+FLC vs Control, P<0.05)
C. albicans biofilm was treated with control (without PAA and FLC), PAA(4μg/mL), FLC(0.5μg/mL), and a combination of PAA(4μg/mL) with FLC(0.5μg/mL). RNA-seq assays were used to determine differential gene expression.

Gene id	Gene Name	FLC vs Control			PAA+FLC vs Control			PAA vs Control		
		log2FoldChange	P value	Significant	log2FoldChange	P value	Significant	log2FoldChange	P value	Significant
CAALFM_C403480CA	hypothetical protein	11.841	0.003773317	yes	11.815	0.033761207	yes	8.518	0.497263972	no
CAALFM_C200560WA	hypothetical protein	12.432	0.001526229	yes	11.708	0.036441333	yes	12.146	0.001062201	yes
CAALFM_C301540WA	hypothetical protein	8.154	0	yes	7.311	3.3605E-106	yes	0.838	1.63966E-05	yes
CAALFM_C406900WA	GST1	4.174	0.011422667	yes	6.675	2.92145E-07	yes	5.129	1.0983E-05	yes
CAALFM_C107040CA	hypothetical protein	5.371	4.38281E-27	yes	6.671	5.91433E-12	yes	1.402	0.126896589	no
CAALFM_C305640WA	hypothetical protein	2.775	0.094415995	no	5.201	5.8369E-06	yes	5.787	1.65338E-12	yes
CAALFM_C500600CA	hypothetical protein	4.753	5.1396E-31	yes	5.055	1.948E-29	yes	0.900	0.081924718	no
CAALFM_C109590CA	LIP10	-0.872	0.549123729	no	4.980	4.32164E-08	yes	3.852	2.47395E-07	yes
CAALFM_C202850WA	UGA6	2.726	3.92846E-17	yes	4.930	1.99696E-36	yes	0.490	0.089590426	no
CAALFM_C400850CA	hypothetical protein	4.703	4.3173E-123	yes	4.737	2.07179E-89	yes	0.233	0.078406545	no
CAALFM_C103870CA	hypothetical protein	3.199	2.03888E-38	yes	4.722	2.15384E-36	yes	0.534	0.010337886	yes
CAALFM_C401010CA	DAG7	2.608	2.18658E-20	yes	4.719	2.77501E-50	yes	-0.587	0.189635673	no

CAALFM_C202220CA	hypothetical protein	3.607	6.53889E-21	yes	4.688	8.51908E-32	yes	-0.683	0.200050748	no
CAALFM_C701060WA	hypothetical protein	4.074	0.017473022	yes	4.663	0.008576083	yes	2.624	0.180386908	no
CAALFM_C701380WA	hypothetical protein	2.687	1.56279E-13	yes	4.664	5.25024E-37	yes	0.258	0.364084068	no
CAALFM_CR08510WA	PGA13	3.184	8.48776E-42	yes	4.640	6.59541E-53	yes	0.551	5.30284E-05	yes
CAALFM_C111710CA	hypothetical protein	2.975	5.04897E-09	yes	4.535	8.56054E-21	yes	1.921	6.28776E-05	yes
CAALFM_C302150CA	ERG6	4.413	4.3592E-100	yes	4.491	9.63333E-95	yes	-0.871	0.017600393	yes
CAALFM_C603500CA	SAP4	2.910	3.97542E-20	yes	4.431	1.33596E-41	yes	0.374	0.183975437	no
CAALFM_C301370CA	PGA44	1.066	0.036381602	yes	4.295	5.22307E-25	yes	0.513	0.072356993	no
CAALFM_C304310CA	hypothetical protein	3.325	8.46222E-24	yes	4.248	1.59249E-22	yes	1.000	0.005623268	yes
CAALFM_C209400CA	ERG24	4.069	5.66184E-88	yes	4.215	1.2357E-44	yes	-0.854	0.004787102	yes
CAALFM_C500470CA	KRE62	0.757	0.403233229	no	4.171	1.09805E-16	yes	0.969	0.048017612	yes
CAALFM_CR03300CA	hypothetical protein	2.124	2.24772E-05	yes	4.122	5.62915E-20	yes	2.659	7.078E-11	yes
CAALFM_C110710CA	hypothetical protein	4.111	1.48524E-56	yes	4.113	1.60544E-24	yes	1.448	7.09438E-08	yes
CAALFM_C208520CA	hypothetical protein	3.655	0.006462764	yes	4.077	0.007634444	yes	2.141	0.124003452	no
CAALFM_C208870CA	PIR1	3.358	2.09938E-77	yes	4.039	3.26096E-50	yes	1.189	2.97667E-13	yes
CAALFM_C303570CA	hypothetical protein	1.131	0.008719429	yes	4.035	2.86977E-27	yes	4.384	7.14557E-44	yes

CAALFM_CR01830CA	PTR2	0.098	0.93746219	no	3.995	0.001980479	yes	-0.089	0.824793178	no
CAALFM_C306660CA	hypothetical protein	4.200	7.4394E-143	yes	3.985	2.04922E-40	yes	0.028	0.339941016	no
CAALFM_CR06580WA	hypothetical protein	0.242	0.849535388	no	3.977	3.32407E-20	yes	1.215	0.000478147	yes
CAALFM_C602210WA	hypothetical protein	2.785	8.24115E-18	yes	3.953	8.74762E-30	yes	0.846	0.005521644	yes
CAALFM_C400450CA	PGA10	4.941	9.38884E-91	yes	3.920	1.38775E-21	yes	0.318	0.205294246	no
CAALFM_C103470CA	hypothetical protein	2.708	1.2985E-14	yes	3.894	5.91561E-27	yes	0.975	0.002606915	yes
CAALFM_C104440WA	hypothetical protein	3.044	0.002705739	yes	3.864	2.18993E-05	yes	1.882	0.061475949	no
CAALFM_C202070WA	SPR3	1.008	0.235189835	no	3.801	2.4511E-10	yes	2.059	0.000109913	yes
CAALFM_C303460CA	hypothetical protein	3.287	1.02515E-28	yes	3.784	7.53446E-30	yes	0.044	0.536181196	no
CAALFM_C105760CA	PGA26	1.217	0.185590731	no	3.775	4.56461E-06	yes	2.267	1.49397E-05	yes
CAALFM_C503050CA	PGA58	2.262	1.07508E-17	yes	3.764	1.38395E-24	yes	0.624	0.004584426	yes
CAALFM_CR06550CA	hypothetical protein	4.021	1.83491E-35	yes	3.753	1.41305E-07	yes	-0.808	0.055985783	no
CAALFM_C403870CA	hypothetical protein	3.312	6.93365E-20	yes	3.724	8.23135E-16	yes	0.227	0.457250399	no
CAALFM_C503520WA	hypothetical protein	3.206	1.40451E-27	yes	3.719	1.06534E-22	yes	0.667	0.023154962	yes
CAALFM_C206530WA	hypothetical protein	0.265	0.680341242	no	3.690	3.74958E-21	yes	0.458	0.077506606	no

CAALFM_CR01850CA	hypothetical protein	2.216	0.001224536	yes	3.592	7.82276E-07	yes	0.772	0.181357401	no
CAALFM_C701560CA	NUP	2.290	1.38818E-10	yes	3.557	2.29721E-19	yes	0.146	0.499732243	no
CAALFM_C501300CA	hypothetical protein	3.068	2.08971E-66	yes	3.546	5.61286E-40	yes	0.311	0.015396865	yes
CAALFM_C402900CA	CRH11	2.983	3.08838E-33	yes	3.544	3.01896E-26	yes	0.289	0.071719536	no
CAALFM_C100800CA	ERG2	3.006	5.33946E-24	yes	3.541	3.60274E-27	yes	-1.196	0.006010732	yes
CAALFM_C206140CA	hypothetical protein	1.361	0.001850763	yes	3.541	1.86846E-18	yes	0.987	0.001534814	yes
CAALFM_C106000WA	hypothetical protein	4.812	2.71483E-85	yes	3.504	6.17725E-21	yes	0.359	0.054147685	no
